# Celastrol: A Promising Agent Fighting against Cardiovascular Diseases

**DOI:** 10.3390/antiox11081597

**Published:** 2022-08-18

**Authors:** Zhexi Li, Jingyi Zhang, Xulei Duan, Guoan Zhao, Min Zhang

**Affiliations:** 1 Department of Cardiology, Life Science Research Center, The First Affiliated Hospital of Xinxiang Medical University, Weihui 453100, China; 2 School of Cardiovascular and Metabolic Medicine & Sciences, King’s College London British Heart Foundation Centre of Research Excellence, London SE5 9NU, UK

**Keywords:** celastrol, obesity, diabetes, atherosclerosis, calcification, heart failure

## Abstract

Cardiovascular diseases (CVD) are leading causes of morbidity and mortality worldwide; therefore, seeking effective therapeutics to reduce the global burden of CVD has become increasingly urgent. Celastrol, a bioactive compound isolated from the roots of the plant *Tripterygium wilfordii* (TW), has been attracting increasing research attention in recent years, as it exerts cardiovascular treatment benefits targeting both CVD and their associated risk factors. Substantial evidence has revealed a protective role of celastrol against a broad spectrum of CVD including obesity, diabetes, atherosclerosis, cerebrovascular injury, calcific aortic valve disease and heart failure through complicated and interlinked mechanisms such as direct protection against cardiomyocyte hypertrophy and death, and indirect action on oxidation and inflammation. This review will mainly summarize the beneficial effects of celastrol against CVD, largely based on in vitro and in vivo preclinical studies, and the potential underlying mechanisms. We will also briefly discuss celastrol’s pharmacokinetic limitations, which hamper its further clinical applications, and prospective future directions.

## 1. Introduction

Cardiovascular diseases (CVDs) are the leading causes of mortality and disability globally, accounting for 17.9 million lives each year, which is equivalent to 31% of total deaths worldwide [1]. Seeking effective therapeutics to reduce the global burden of cardiovascular disorders has become increasingly urgent. In the past decade, natural compounds—especially Chinese herbal extracts and their chemical monomers—have attracted extensive attention as alternative treatments for CVD [2,3]. One promising drug candidate that has been in the spotlight in recent years is celastrol [2,3]. Celastrol is an orange-coloured triterpene derived from the roots of *Tripterygium wilfordii* Hook F (TWHF, also known as “Thunder God Vine” or “lei gong teng”), originating from China. Molecularly, celastrol is a pentacyclic triterpenoid that resides in the family of quinone methides [4], with a chemical structure of (9β, 13α, 14β, 20α)-3-hydroxy-9, 13-dimethyl-2-oxo-24,25,26-trinoroleana-1(10), 3, 5, 7 tetraen-29-oic acid. Historically, the root extracts, despite its toxicity and detriment with high-dose administration clinically [5,6], have long been recognised as a Chinese traditional therapeutic for autoimmune disorders such as rheumatoid arthritis, multiple sclerosis and systemic lupus erythematosus. The root pulp possesses various active phytochemicals including terpenoids, alkaloids and steroids, whilst celastrol is the most abundant bioactive molecule.

Substantial studies have reported that celastrol possesses a variety of therapeutic potentials in a diverse range of disease states including anticancer and neuroprotection [7,8]. In recent years, emerging evidence from both in vitro and in vivo preclinical research strongly indicates celastrol as a promising agent fighting against CVD through complicated and interlinked mechanisms such as direct protection against cardiomyocyte hypertrophy and death, and indirect beneficial effects against oxidation and inflammation. Celastrol, however, has some pharmacokinetic limitations, including its limited water solubility, low oral bioavailability, and potential intolerability in vivo, which hamper its further clinical applications. Thus, to gain a deep understanding about the protection of the plant-derived compound celastrol against CVD, this review will: (a) mainly summarize the therapeutic value of celastrol in CVD, including obesity, diabetes, atherosclerosis, cerebrovascular injury, calcific aortic valve disease and heart failure, and their possible underlying mechanisms; (b) briefly discuss the associated limitations and future perspectives.

## 2. Celastrol against Metabolic Disorders

### 2.1. Obesity

**Figure 1:**
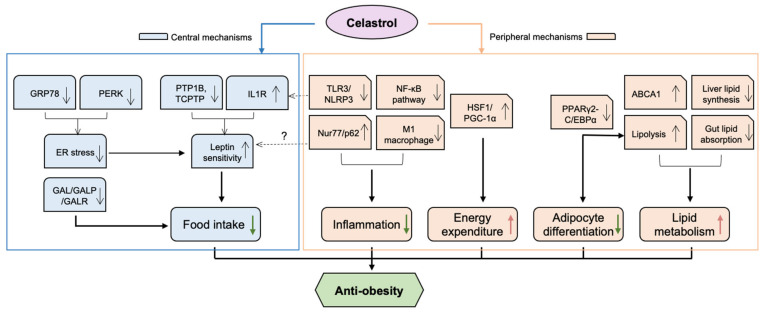
**The potential mechanisms underlying the anti-obesity effect of celastrol.** Celastrol protects against obesity through both central and peripheral mechanisms. Mainly, in the hypothalamus, celastrol enhances leptin sensitivity by reducing ER stress via inhibition of PERK phosphorylation and possible GRP78, as well as activating IL1R and inhibiting PTP1B/TCPTP pathways. Celastrol can also attenuate food intake of obese mice by downregulating the levels of GAL, GALR1 and GALR3 in the brain. In terms of peripheral mechanisms, celastrol alleviates inflammatory responses by inhibiting both TLR3/NLRP3 inflammasome and NF-KB pathway. Celastrol also reduces inflammation and improves metabolic disorders by increasing Nur77/p62-mediated mitophagy. Celastrol activates HSF1/PGC1α, which results in the enhancement of energy expenditure and mitochondrial function in adipose tissues and muscles. Celastrol inhibits adipocyte differentiation and increases lipid metabolism by blocking PPARγ2- or C/EBPα-mediated transcriptional activity. Moreover, celastrol improves lipid metabolism by upregulating ABCA1, by reducing liver lipid synthesis, and by inhibiting gut lipid absorption by modulating gut microbiota. ABCA1—ATP-binding cassette transporter A1; C/EBPα—CCAAT/enhancer binding proteins-α; GAL—galanin; GALP—galanin-like peptides; GALR—galanin receptor; GRP78—78 kDa glucose-regulated protein; HSF1—heat shock factor 1; IL1R—interleukin-1 receptor; NF-κB—nuclear factor-κB; NLRP3—nucleotide-binding oligomerization domain—leucine-rich repeat—and pyrin domain containing 3; PGC-1α—peroxisome proliferator-activated receptor γ coactivator-1α; PERK—Protein kinase R-like ER kinase; PPARγ2—peroxisome proliferator-activated receptors-γ2; PTP1B—protein tyrosine phosphatase 1B; TCPTP—T-cell protein tyrosine phosphatase; TLR3—Toll-like receptor 3. Red arrows indicate “increase”, green arrows indicate “decrease”. Question marks mean “possible action and effect”.

Obesity, classified as a body mass index (BMI) above 30 kg/m^2^, has grown to epidemic proportions, affecting 650 million adults worldwide [9]. Especially during and post COVID-19, various factors, including virus-induced inflammation and change in life style, have contributed greatly to aggravating obesity, particularly in women [10]. Obesity is a risk factor for a wide range of noncommunicable diseases and conditions, including type 2 diabetes mellitus (T2DM), chronic kidney diseases (CDK), long COVID [11], and various CVD [12]. Factors contributing to obesity are diverse; nonetheless, it is commonly accepted that the main cause of obesity is long-term energy intake/expenditure imbalance. Increasing evidence supports that celastrol ameliorates obesity via both central and peripheral mechanisms, influencing food intake, energy expenditure, lipid metabolism, adipocyte differentiation and inflammation. These mechanisms are interrelated, demonstrating celastrol’s pleiotropic modes of action against metabolic disorders (Figure 1).

#### 2.1.1. Anti-Obesity Effect of Celastrol through Central Mechanisms

##### A Leptin Sensitiser

Leptin is an anorexigenic adipocyte-derived hormone, which predominately acts on the central nervous system (CNS) to mediate satiety, and subsequently increases food intake and body mass [13]. Leptin interacts with leptin transmembrane receptors (LEP-Rs), particularly the isoform LEP-Rb expressing on pro-opiomelanocortin (POMC) neurons, which are located in the arcuate nucleus (ARC) of the hypothalamus and nucleus tractus solitarius of the brainstem, to regulate appetite and metabolism [14,15]. This binding triggers a cascade of reactions and an increase in the synthesis and release of anorexigenic hormones, including α-melanocyte-stimulating hormone (α-MSH), and inhibits orexigenic neuropeptide Y (NPY)/agouti-related protein (AgRP) pathways, ultimately leading to a decrease in food intake [16]. Paradoxically, however, plasma leptin is found to be higher in obese individuals, suggesting a state of leptin resistance that obstructs leptin’s sustained therapeutic properties [17,18]. Thus, to develop an effective obesity therapy based on leptin, the prevention of leptin resistance is a vital challenge. In a pioneering study, Liu and co-workers (2015) uncovered that celastrol is a leptin sensitiser and exerts robust anti-obesity properties. By employing an in silico screening approach, the team identified celastrol as a potent leptin sensitiser. Both i.p and oral celastrol administration decreased the mouse body weight by up to 45% in hyperleptinemic diet-induced obese (DIO) mice by suppressing food intake [13]. Celastrol also potentiates the effect of leptin by augmenting the hypothalamic LEP-R-STAT3 phosphorylation, an effect that could be further enhanced by leptin injection. Importantly, celastrol does not have any effect in leptin-deficient db/db or ob/ob mice, the observations validating celastrol as a leptin sensitiser, which provides evidence that celastrol holds potential in treating leptin-resistant obesity. Of note, with the use of a higher dosage of celastrol (0.5 mg/kg), mild body weight reduction was still observed in LEP-R-null mice, which raises the possibility that celastrol may act through an unknown mechanism other than LEP-R-dependent signalling to promote weight loss [19]. In addition, other studies reported that celastrol protected mice from weight gain without affecting their food intake [19,20]. This inconsistency is probably due to different mouse models and different dosages of celastrol being used. Of note, celastrol may also reduce body weight independent of hyporexia by enhancing energy expenditure through peripheral mechanisms (see below).

To further investigate the genes and pathways of leptin sensitisation induced by celastrol, it was later identified interleukin-1 receptor 1 (IL1R1) as the mediator of leptin sensitivity in celastrol-treated DIO, lean, and db/db mice by analysing the hypothalamic transcriptomes. DIO mice with functioning IL1R1 (Il1R1^+/+^) had reduced food intake and body weight after celastrol treatment, whereas IL1R1 deficient (Il1R1^−/−^) DIO mice did not. Administering IL1R1 inhibitor (anakinra) to celastrol-treated mice also attenuated the anti-obesity effects of celastrol [21]. IL1R1 is the major receptor of the IL-1 family, which has diverse functions in body homeostasis during immune responses and other processes. Evidence also documents that following administration of IL-1 to murine models, mRNA and protein levels of leptin increase [22]. This positive correlation between IL-1 and leptin is explained by leptin being a pro-inflammatory adipokine, as Lipocalin-2 (LCN2), a potent bacteriostatic agent, was the most strongly upregulated gene in the hypothalamus of celastrol-treated DIO mice. However, genetic LCN2 deficiency does not accelerate diet-induced obesity, nor does it alter impaired glucose homeostasis or hepatic function [23]. This suggests that celastrol-mediated anti-obesity effects do not involve LCN2, and the exact mechanism remains unelucidated.

Moreover, celastrol increases leptin sensitivity through the inhibition of leptin-negative regulators protein tyrosine phosphatase 1B (PTP1B) and T-cell PTP (TCPTP) in the ARC of the hypothalamus, where PTP1B and TCPTP negatively modulate leptin signalling by dephosphorylating the downstream effectors JAK2/STAT3 [24]. The in vivo genetic deletions of PTP1B and/or TCPTP resulted in a complete abolishment of celastrol’s weight-lowering effects. The following in vitro study further revealed that celastrol inhibits PTP1B and TCPTP and restores CNS leptin sensitivity via reversible non-competitive allosteric binding to the catalytic domain. However, Pfuhlmann and colleagues (2018) disagree with the notion that PTP1B has a role in celastrol-mediated leptin sensitivity restoration [25]. They reported that global PTPB1 KO did not diminish the weight-reducing activity of celastrol. Given that PTP1B and TCPTP share high sequence homology, celastrol could plausibly inhibit TCPTP to take effect instead; however, the effect of celastrol on TCPTP alone needs further investigation.

Interestingly, systemic administration of celastrol produces body weight-reducing effects independent of melanocortin 4 receptor (MC4R) [19]. This could shed a light on potential treatments for obese individuals with a genetic MC4R deficiency, accounting for around 4% of morbid obesity (BMI > 40 kg/m^2^) [26].

##### An Endoplasmic Reticulum (ER) Stress Suppressor

Obesity is closely associated with hypothalamic endoplasmic reticulum (ER) stress responsible for leptin resistance and perturbation of energy homeostasis. Disruption of ER homeostasis triggers a complex intracellular signalling cascade termed the unfolded protein response (UPR). Protein kinase R-like ER kinase (PERK) is one of the transmembrane proteins that mediate UPR, and phosphorylated PERK contributes to ER stress and serves as one of the ER stress markers [27]. Celastrol reduces ER stress in the hypothalamus by reducing PERK phosphorylation and sarco/endoplasmic reticulum Ca^2+^-ATPase isoform 2b (SERCA2b) protein levels [13]. POMC neurons in the ARC of the hypothalamus have been found to be key nutrient sensors involved in metabolic regulation [28]. He et al. identified that it is the POMC-specific PERK reduction which is responsible for the celastrol-induced weight loss and restoration of leptin sensitivity [29]. In addition, the 78 kDa glucose-regulated protein (GRP78) acts as a complete ER chaperone and is critical in the differentiation of preadipocytes into adipocytes [30]. It was recently reported that celastrol covalently binds and conjugates to the residue Cys^41^ of GRP78, and consequently reduces ER stress, inflammation, and lipid accumulation in palmitate-challenged macrophages [31]. However, the role of celastrol in hypothalamic ER stress needs to be further explored, since the expression levels of other ER stress-related genes, such as Xbp1s and CHOP, did not alter following celastrol treatment in lean, DIO, or MC4R-null mice [19].

##### Downregulation of Galanin (GAL)

The involvement of GAL, a 29/30-amino-acid orexigenic neuropeptide, has been recently identified in feeding behaviour regarding food intake. GAL also interacts with other appetite-regulating peptides, including NPY and leptin, to overall control feeding [32]. A recent study showed that celastrol decreased the levels of GAL, galanin-like peptides (GALP), and galanin receptor (GALR) 1 and 3 in the hypothalamus to attenuate fat intake and weight gain in DIO mice [33]. This finding suggests a possible role of celastrol in the central regulation of food intake by interfering with the GAL system, though further studies are required to unravel the underlying mechanism.

#### 2.1.2. Anti-Obesity Effect of Celastrol through Peripheral Mechanisms

##### Modulation of Lipid Metabolism

Celastrol play an anti-obesity role not only through anorexia, but also by increasing energy expenditure and promoting lipid metabolism. Celastrol was reported to suppress body weight and reduce high-fat mediated cardiovascular damage by reducing oxidative stress and improving lipid metabolism, namely by upregulating ATP-binding cassette transporter A1 (ABCA1) expression [34]. Consequently, the plasma levels of cholesterol, triacylglycerol, LDL-c, apolipoprotein B, and malondialdehyde (MDA), as well as NADPH oxidase (NOX) activity, were alleviated after oral celastrol treatment in high-fat emulsion (HFE)-fed rats, suggesting improved lipid metabolism. Furthermore, lipidomic profiling revealed that celastrol promotes the metabolism of diverse classes of lipids by downregulating mRNAs encoded by hepatic genes associated with lipid biosynthesis and catabolism, such as *Cers6* and *Acer2* [35]. The effect of celastrol on lipid metabolism is farnesoid X receptor (FXR)-dependent; nevertheless, further investigation on the mechanism by which celastrol modulates FXR signalling to regulate lipid metabolism is needed.

Intriguingly, celastrol can inhibit intestinal lipid absorption and increase faecal lipid excretion by modulating gut microbiota composition. Dietary celastrol downregulates the expression of intestinal lipid transporters, such as apolipoprotein B-100 (ApoB), very-long-chain acyl-CoA synthetase 2 (FATP2), and long-chain fatty acid transport protein 4 (FATP4) in DIO mice. The effect can be mimicked by antibiotics or faecal microbiota transplantation, suggesting a role of resetting the gut microbiota profile in the anti-obesity effect of celastrol [36]. In addition, it was shown that celastrol could improve the diversity of gut microbiota, in particular, with an increased ratio of Bacteroidetes to Firmicutes [37]. These studies also observed that celastrol reduced the body weight, yet did not affect food intake in mice and rats fed HFD, suggesting celastrol’s anti-obesity effect is multifactorial in addition to leptin sensitising [36,37]. Moreover, a recent study combining the shotgun metagenomic sequencing profile of the gut microbiome and pseudotargeted metabolomics analysis further provided the potential mechanistic connection among the alterations in gut microbes, serum metabolome, and celastrol-induced weight loss [38].

##### A Heat Shock Factor 1 (HSF1) Activator

HSF1 is a classic transcription factor induced by multiple stimuli, including heat shock, cold temperature, oxidative and mechanical stresses. HSF1 regulates energy expenditure through the activation of a peroxisome proliferator-activated receptor γ coactivator-1α (PGC-1α)-dependent metabolic program in adipose tissues and muscles [39]. Celastrol activates HSF1, as a result, increasing energy expenditure, enhancing mitochondrial function in fat and muscle, as well as protecting against obesity, insulin resistance, and hepatic steatosis in mice fed HFD; the effects were totally abolished in HSF1 knockout mice [20].

##### Inhibition of Adipocyte Differentiation

Increased adipocyte differentiation has been implicated in the pathophysiology of obesity [40]. Choi et al. demonstrated that celastrol inhibits adipocyte differentiation and promotes lipolysis in cultured 3T3-L1 adipocytes by downregulating peroxisome proliferator-activated receptors-γ2 (PPARγ2) and CCAAT/enhancer binding proteins-α (C/EBPα) [41]. PPARγ and C/EBPα are two pivotal transcription factors and are closely involved in adipocyte differentiation [42]. Consistent with this, celastrol was found to exert an inhibitory effect on adipogenic differentiation and lipid accumulation of human adipocyte-derived stem cells (hADSCs), in a dose, time, and duration-dependent manner, and the inhibition was also mediated by PPARγ and C/EBPα [43]. Furthermore, the findings also demonstrate that celastrol suppresses differentiation of hADSCs into the osteogenic and chondrogenic lineages and inhibits the multilineage differentiation capacity of ADSCs, indicating an intimate regulatory role of celastrol in cell differentiation [43].

##### Anti-Inflammatory Activities

It is well documented that chronic inflammation contributes to obesity development, and pro-inflammatory monocytes such as macrophages are often accumulated in adipose tissues in obese individuals [44]. It is well known that, in response to stimuli such as pathogenic compounds or various cytokines, macrophages possess the ability to polarise towards pro- or anti-inflammatory phenotypes, classic (M1) and alternative (M2), respectively, with each phenotype exerting distinct cellular functions [45]. M1 macrophages deteriorate inflammation and promote adipose tissue dysfunction and insulin resistance in obesity [46]. Celastrol exerts its weight-reducing properties by suppressing macrophage M1 polarisation via multiple pathways: regulation of MAP kinases (e.g., ERK1/2, p38, JNK), inhibition of nuclear translocation of nuclear factor-κB (NF-κB) p65 subunit, and activation of the nuclear factor erythroid 2–related factor 2 (Nrf2), subsequently inducing haem oxygenase-1(HMOX-1) expression in DIO mice [47]. The ability of celastrol to skew macrophage polarization to the anti-inflammatory M2 phenotype was further validated using PEG-PCL nanoparticles-loaded celastrol [48]. Likewise, Abu Bakar et al. reported celastrol diminished mRNA expression of pro-inflammatory macrophage M1 phenotype in adipose tissue of HFD rats by downregulating NF-κB activity [49]. Taken together, celastrol exhibits anti-inflammatory activities by modulating macrophage polarisation to promote weight loss and lipid accumulation.

Low-grade inflammation in white adipose tissue (WAT) involving increased IL-1β levels contributes to the pathology of obesity [50]. Nucleotide-binding oligomerization domain, leucine-rich repeat, and pyrin domain containing three (NLRP3), regulated by Toll-like receptor (TLR3), is an innate immune sensor triggered by pathogen infection that controls inflammasome production. Activated NLRP3 promotes inflammasome production and subsequently triggers the generation and release of pro-inflammatory cytokines, including IL-1β [51]. Celastrol reduces macrophage infiltration in liver and adipose tissues and inhibits TLR3/NLRP3-dependent inflammasome activation pathways, inhibiting metabolic inflammation, increasing adipose thermogenesis and ultimately enhancing body energy expenditure as a result [52]. Considering the involvement of neural IL1R1 in celastrol-dependent anti-leptin resistance, as discussed above [21], it is reasonable to postulate that IL1R1 is complicatedly implicated in the protection of celastrol against obesity through both central and peripheral mechanisms.

The protective effects of Nur77, an orphan member of the nuclear receptor superfamily, have been highlighted in CVDs and metabolic disorders, including obesity, owing to its potent anti-inflammatory properties [53,54]. Celastrol binds directly to Nur77 and induces autophagy to ameliorate inflammation by promoting mitochondrial ubiquitination via Nur77/tumour necrosis factor receptor-associated factor 2 (TRAF2) signalling [55]. This study unravels that celastrol interacts with a Nur77-dependent pathway to reduce inflamed mitochondria and ultimately promotes weight loss. Notably, hypothalamic Nur77 facilitates STAT3 acetylation and consequently modulates the downstream gene expression to enhance the sensitisation of leptin; the result suggests the important participation of Nur77 in leptin central control of food intake [53], though the role of celastrol in this Nur77-mediated leptin sensitisation is yet to be explored. The team further pursued the mechanism of Nur77 in celastrol-induced mitophagy; their finding shows that p62, one of the most well-characterized selective autophagy receptors, is required for Nur77-dependent mitophagy, and that Nur77-p62 condensates facilitate celastrol-induced mitophagy [56].

AMP-activated protein kinase (AMPK) has a central role in mitochondrial homeostasis and cellular energetic stress response. AMPK phosphorylates PGC-1α as well as indirectly regulates PGC-1α involving sirtuin 1 (SIRT1) [57]. Sirtuin 1 (SIRT1), an NAD^+^-dependent histone deacetylase that is induced by calorie restriction, participates in the regulation of various metabolic cellular functions involving mitochondrial biogenesis, lipid metabolism, and inflammation by activating PPARγ and PGC-1α [58]. Celastrol improves muscle mitochondrial functions in the HFD-fed rats by upregulating AMPK/SIRT1 activities and therefore increasing levels of PGC-1α deacetylation and NAD^+^/NADH ratios [49].

### 2.2. Diabetes Mellitus

Diabetes mellitus unequivocally exacerbates CVD as a major risk factor, as the core hallmarks of type II diabetes (T2D) contribute to endothelial dysfunction and thermogenesis acceleration, such as insulin resistance and impaired glucose tolerance [59,60]. Despite the remarkable progress that has been made in T2D drug discovery over the last two decades, the associated adverse effects, including hypoglycaemia, heart failure, and osteoporosis, limit their clinical use [61]. Thus, it is imperative to develop novel anti-diabetic agents with minimised undesirable effects to combat T2D.

Insulin resistance and insulin deficiency are the common pathologies of T2D; thus, reversing insulin resistance is one of the therapeutic strategies used to treat T2D [62]. Abundant evidence has linked insulin resistance to adipose tissue inflammation, in which NF-κB pathway plays a central role [63,64]. It was reported that celastrol as an NF-κB inhibitor could improve insulin resistance, glucose control, and oxidative stress, accompanying improved common T2D complications such as diabetic nephropathy and renal and kidney dysfunction [65]. Moreover, celastrol reverses palmitic acid-induced insulin resistance by activating Toll-like receptor (TLR4)/myeloid differentiation factor 2 (MD2)/NF-κB signalling [66]. Celastrol directly binds to MD2 to inhibit TLR4/NF-κB activation [67]. Insulin resistance is established to correlate with elevated free fatty acids (FFAs) in the plasma, as FFAs induce inflammation involving TLR4/NF-κB signalling [68]. Intriguingly, hypothalamic NF-κB activation was found to mediate infection-induced anorexia and weight loss via direct activation of POMC [69]. NF-κB also acts as a downstream signalling pathway of leptin in hypothalamus [69]. These findings imply the complicated actions of the inflammatory NF-κB pathway in metabolic disorders.

Apart from the direct anti-obesity property resulting from inhibition of the orexigenic GAL, celastrol modulates glucose uptake and consumption [33]. In 3T3-L1 cells, celastrol treatment upregulates PGC-1α/glucose transporter type 4 (GLTU4) expression in adipocytes and skeletal muscle via AKT and P38 MAPK, consequently improving insulin resistance, as well as inhibiting gluconeogenic activity through a CREB/PGC-1α pathway [33]. Another study shows that celastrol protects against palmitate-induced insulin resistance in C2C12 myotubes through PI3K-Akt activation, accompanied by improved mitochondrial function and glucose uptake [70]. In vivo, celastrol administration resulted in the alleviation of glucose intolerance and insulin insensitivity assessed by intra-peritoneal glucose tolerance test (GTT) and insulin tolerance test (ITT) in DIO mice, respectively [47].

## 3. Celastrol against Atherosclerosis

Atherosclerosis, the underlying pathophysiology for major cardio- and cerebrovascular diseases, involves chronic inflammation of the arterial wall as well as redox imbalance, and is highly associated with disturbed lipid metabolism. This results in macrophages accumulating in the subendothelial space and phagocytosing modified lipoproteins to give rise to pro-inflammatory factor secreting foam cells. Cytokines such as vascular endothelial growth factor (VEGF) and tumour necrosis factor-α (TNF-α) stimulate the migration of vascular smooth muscle cells (VSMCs) into the intima, where they also uptake lipids, develop lesions and narrow the arteries further. Plaques can rupture to stimulate the formation of blood clots, possibly resulting in myocardial infarction (MI) or stroke [71]. Growing evidence suggests that celastrol holds promise as a therapeutic for the progression of atherosclerosis into more serious cardiovascular and cerebrovascular events.

Various studies reveal that celastrol reduces the plaque size, partially owing to its antioxidant property. In vitro, celastrol inhibits oxidized low-density lipoprotein (oxLDL)-induced lectin-like oxidized low-density lipoprotein receptor-1 (LOX-1) expression and the subsequent reactive oxygen species (ROS) production in macrophages. In an apolipoprotein E knockout (apoE^−^^/^^−^) mouse model fed a high-fat/high-cholesterol (HFHC) diet, it was verified that celastrol could attenuate atherosclerotic plaque development by lowering LOX-1 [72]. Moreover, celastrol was found to reduce the size of plaque in the arterial wall by decreasing the LDL-cholesterol (LDL-C) level in the serum and the expression of VEGF in a rabbit carotid atherosclerosis model [73]. In a recent study using a novel delivery system for celastrol by employing nanocarriers to reduce its cytotoxicity, it was reported that this encapsulation of celastrol into poly (ethylene glycol)-b-poly (propylene sulphide) (PEG-b-PPS) micelles resulted in a reduction in the atherosclerotic plaque size by decreasing TNF-α secretion and inhibiting NF-κB after LPS stimulation of macrophages, as well as anti-inflammatory effects in the LDL receptor-deficient (Ldlr^−/−^) mouse model [74]. This provides a novel strategy for lowering celastrol’s cytotoxicity for the potential clinical treatment of atherosclerosis.

Celastrol counteracts the THP-1-derived macrophage secretion of the adipokine resistin, a main resource of human resistin [75,76]. Interestingly, celastrol inhibits resistin-induced human aortic smooth muscle cells (SMC) migration by disturbing the SMC-intimal collagen matrix interaction by disturbing the Toll-like receptor 4 (TLR-4) pathway [77]. Celastrol also reduces angiotensin II (Ang II)-induced ROS production and senescence of VSMCs by enhancing autophagy [78]. Likewise, celastrol triggers VSMC autophagy and inhibits lipid accumulation in VSMCs by upregulating ABCA1 expression through the activation of liver X receptor α (LXRα) [79].

Endothelial cell dysfunction and death is the pivotal step in the initiation of atherosclerosis. It was reported that celastrol protects Ang II-stimulated endothelial cell apoptosis by reducing NOX2-mediated ROS production through the activation of Nrf2/ERK1/2 signalling [80]. Interestingly, celastrol may also enhance endothelial progenitor cell (EPC) function via the upregulation of HSP32 (also known as haem oxygenase-1, HMOX-1), and improve vascular function by suppressing EPC apoptosis via a mechanism involving integrin-linked kinase (ILK) and its downstream effectors Akt and GSK-3β [81].

Platelets play important roles in contributing and possibly accelerating atherosclerosis [82]. Hu and colleagues (2009) reported that celastrol inhibits platelet activation. The in vitro study showed that celastrol inhibited human platelet activation marker P-selectin after platelet activation by its agonists, including adenosine-5-diphosphate (ADP), thrombin, and phorbol 12-myristate 13-acetate (PMA) [83]. Celastrol partially prevents ADP-induced platelet adhesion on fibrinogen and inhibits ADP-stimulated platelet fibrinogen binding in mice [83].

## 4. Celastrol against Cerebrovascular Injury

Inflammation and excess ROS production extensively contribute to cerebrovascular injury. Celastrol reduces inflammatory damage and oxidative stress, which may provide a potential treatment option for cerebrovascular diseases.

In the permanent middle cerebral artery occlusion (MCAO) rat model, celastrol administration after acute stroke showed a spectrum of protective effects, including reducing neurological deficit, brain water content and infarct sizes, as well as screwing M2 microglia phenotype polarisation [84]. Additional studies have discovered that celastrol downregulates the expression of p-JNK, p-c-Jun and NF-κB. M2 microglia polarisation is promoted after celastrol treatment via growth stimulation expressed gene 2 (ST2)/IL-33 activation, which is neuroprotective after acute ischaemia [84,85]. The findings of the cell model employing oxygen-glucose deprivation (OGD) in primary neuron–microglial co-cultures further substantiate that celastrol activates ST2/IL-33 axis and consequently triggers M2 phenotype polarisation in microglia/macrophages, thereby protecting neurons from ischaemic injury and inhibiting neuronal apoptosis [85].

Celastrol was also reported to protect against cerebral ischaemia/reperfusion (I/R) injury. In response to MCAO followed by reperfusion in rodent models, celastrol reduces cerebral infarct volumes and apoptosis, and can reverse cerebral I/R injury-induced alteration of phosphatidylcholine, phosphatidylethanolamine and sulfatide, suggesting the regulatory impact of celastrol on lipidomics may partially account for its neuroprotective effects [86]. A recent study indicates that celastrol may provide neuroprotection in cerebral I/R injury through its metabolic regulating properties. Celastrol treatment attenuates I/R-induced hyperglycolysis through the inhibition of hypoxia inducible factor-1α (HIF-1α)/pyruvate dehydrogenase kinase1 (PDK1) pathway [87]. Moreover, bioinformatics analysis through RNA sequencing (RNA-Seq) suggests that inflammation-related signalling pathways play vital roles in the protection of ischemic stroke by celastrol [88]. A study utilising transient global cerebral ischaemia reperfusion (tGCI/R) rats also provides proof that celastrol attenuates neuroinflammation, decreases neuronal apoptosis and oxidative stress by inhibiting high mobility group box protein 1 (HMGB1)/NF-κB signalling pathway [89]. Further investigation with the help of quantitative chemical proteomics technology found that celastrol directly binds to and blocks the activation of HMGB1 to promote neural survival against I/R challenge by targeting heat shock protein 70 (HSP70) and NF-κB p65 in OGD and cerebral I/R injury models [90]. Aside from the anti-inflammatory effects, celastrol displays antioxidant properties in tGCI/R rats by increasing antioxidative markers including glutathione (GSH), superoxide dismutase (SOD), and catalase. Simultaneously, celastrol also partially inhibits glial activation and proliferation upon tGCI/R insult [89].

Furthermore, celastrol improves cerebral artery constriction. For the first time, the data from North et al. suggest celastrol evokes endothelium-independent middle cerebral artery (MCA) dilation by mediating with voltage-gated (KV) and calcium- and voltage-gated potassium channel of large conductance (BK) K+ channels, which widens the potential therapeutic use of celastrol as a cerebrovascular dilator under particular conditions such as impaired BK channel and/or endothelium [91].

Additionally, celastrol was shown to protect against early brain injury (EBI) after subarachnoid haemorrhage (SAH) [92]. In the rat SAH endovascular perforation model, celastrol improves neurological function, reduces brain swelling caused by SAH, and T2 lesion volume and ventricular volume. This neuroprotective effect of celastrol is achieved via inhibition of matrix metalloproteases-9 (MMP-9) and neuroinflammation, alleviation of the blood–brain barrier disruption, and prevention of the receptor interacting protein kinase-3/mixed lineage kinase domain-like protein (RIP3/MLKL)-mediated necroptosis.

## 5. Celastrol against Valvular and Vascular Calcification

Ectopic calcification has a variety of forms, depending on where the mineral is deposited. Both intimal calcification and medial calcification are two traditional classes, but the deposition can also be found in the valves of the heart [93]. Valvular and vascular calcification is one of the prominent risk factors for CVD, and there remains a lack of effective treatment options [94]. Calcific aortic valve disease (CAVD) can lead to valve hardening and aortic stenosis, and vascular calcification can result in vasodilation dysfunction and impaired vascular resistance, both of which contribute to heart failure. Both valvular and arterial calcification occur via active processes, though they may be regulated by distinct disease-driving mechanisms. Under the stimulation of high calcium and phosphate levels, inflammatory cytokines, as well as oxidative stress, aortic valvular interstitial cells (AVICs) or VSMCs mediate pathogenic progress of valvular and vascular calcification by transdifferentiating into osteoblastic-like cells and secreting matrix proteins. This process of osteogenic-like differentiation of quiescent AVICs and VSMCs shares some common signalling pathways, such as runt-related transcription factor 2 (Runx2), transforming growth factor β (TGF-β), and Wnt signalling family [95,96]. However, the translation from preclinical experiments to patients is poor; therefore, there is an urgent clinical need to identify other therapies [97].

Increased oxidative stress is critically implicated in the trans-differentiation of AVICs to an osteoblastic phenotype, as exogenous ROS promotes AVIC differentiation [98,99]. Similarly, elevated ROS production in VSMCs leads to the release of extracellular vesicles (EVs) which promote phenotype switching and, ultimately, calcification [100]. Therefore, inhibition of ROS production will be a reasonable approach to alleviate CAVD and vascular remodelling and calcification. However, the contribution of ROS depends on their types, levels, and intracellular sources. NADPH oxidase-2 (NOX2) belongs to the NOX family proteins, which are known as the “professional” cellular ROS-generating enzymes. NOX enzymes are pivotal ROS sources in cardiovascular systems, functioning as electron transporters across biological membranes and catalysts for the reduction of molecular oxygen to generate ROS [101,102]. NOX2 is widely expressed in the heart and vessels, which not only involves in physiological functions but also in the pathogenesis of a range of cardiovascular diseases such as cardiac hypertrophy, fibrosis, inflammation, metabolic disorders, and atherosclerosis [103,104]. Importantly, the expression levels of NOX2 and its regulatory subunits p47^phox^, p22^phox^ and p67^phox^ are increased in high calcium-induced CKD rats, human aortic smooth muscle cells, and rabbit calcified aortic valves, indicating that NOX2 may provide a potential therapeutic target in alleviating vascular and valvular calcification [99,105,106].

**Figure 2:**
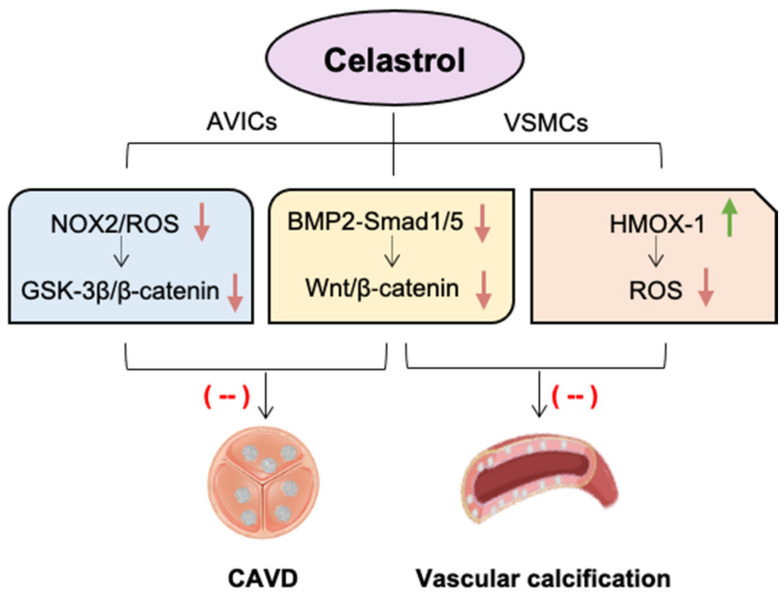
**The beneficial effects of celastrol against valvular and vascular calcification.** Celastrol, as a natural potent NOX2 inhibitor, alleviates calcific aortic valve disease (CAVD) by blocking glycogen synthase kinase 3 beta (GSK3 β)/β-catenin pathway in aortic valve interstitial cells (AVICs). Likewise, celastrol reduces reactive oxygen species (ROS) production and oxidative stress by upregulating haem oxygenase-1 (HMOX-1) in vascular smooth muscle cells (VSMCs). Celastrol also has the capacity to alleviate both valvular and vascular calcification via inhibition of bone morphogenetic protein 2 (BMP2)-Smad1/5 and Wnt/β-catenin signalling. Green arrow indicates “increase”, red arrows indicate “decrease”.

Celastrol is a natural compound as a NOX modulator that dose-dependently inhibits NOX1, NOX2, NOX4, and NOX5 at low concentrations with a higher Hill coefficient and lower IC50 values for NOX2, indicating higher potency against NOX2 [4,107]. Our recent studies showed that NOX2 levels were significantly increased in both human and rabbit calcific aortic valve tissues. In vitro, NOX2 is markedly induced in cultured porcine AVICs stimulated with osteogenic medium, accompanied by the elevated production of ROS and the increased formation of calcium nodules. This phenomenon can be significantly attenuated by celastrol treatment or the knockout of endogenous NOX2; the effect is likely through the inhibition of glycogen synthase kinase 3 beta (GSK3 β)/β-catenin pathway in AVICs. In a rabbit CAVD model induced by a high-cholesterol diet plus vitamin D2, celastrol effectively reduces aortic valve fibrosis and calcification, mitigates aortic stenosis and protects heart function (Figure 2) [108].

The beneficial effect of celastrol on valve calcification was also observed in the chronic kidney disease (CKD) mouse model induced by an adenine diet [109]. Interestingly, the same study revealed the protection of celastrol against high calcium-induced arterial calcification as well, indicating the capability and efficacy of celastrol in alleviating both valvular and vascular calcification [109]. In vitro, celastrol decreases the expression of osteogenic markers Runx2 and osteopontin in cultured porcine AVICs and human aortic VSMCs via inhibition of bone morphogenetic protein 2 (BMP2)-Smad1/5 and Wnt/β-catenin signalling [109]. It was recently reported that celastrol could effectively attenuate calcium/phosphate-induced rat VSMC calcification in vitro, calcification of arterial rings of rat and human ex vivo, and aortic calcification in CKD rats and vitamin D3-overloaded mice in vivo [110]. Mechanistically, celastrol reduces ROS production and oxidative stress by upregulating HMOX-1 in rat VSMC, since a specific HMOX-1 inhibitor, zinc protoporphyrin-9 (ZnPP-9), could eliminate this protective effect of celastrol [110].

## 6. Celastrol against Heart Failure

**Figure 3:**
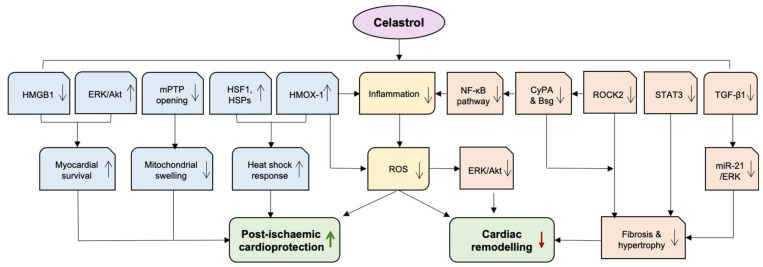
**The cardiac protection of celastrol.** Celastrol exhibits beneficial effects on adverse cardiac remodelling and post-ischemic protection, mainly attributable to its anti-oxidative and anti-inflammatory capacity outlined in the main text. AKT—protein kinase B; Bsg—basigin; CyPA—Cyclophilin A; ERK—extracellular signal-regulated kinases; HMGB1—high mobility group box protein 1; HMOX-1—haem oxygenase-1; HSF1—Heat shock factor 1; HSR—heat shock response; mPTP—mitochondrial permeability transition pore; NF-κB—nuclear factor-κB; ROCK—RhoA/Rho-kinase; ROS—reactive oxygen species; STAT3—Signal transducer and activator of transcription 3; TGF-β—transforming growth factor β. Green arrow indicates “increase”, red arrow indicates “decrease”.

Heart failure (HF) is the principal cause of hospitalization in patients over the age of 65, affecting 64.3 million globally and 4 million people in the UK [111]. Adverse cardiac remodelling in response to injury especially after MI remains the major driver of HF. Oxidative stress and inflammation are critically implicated in the pathophysiology of HF and its predisposing conditions, such as hypertension [112,113]. Emerging evidence indicates that celastrol alleviates the occurrence and progression of HF in experimental models, and this is mainly attributable to its anti-oxidative and anti-inflammatory capacity (Figure 3).

### 6.1. Mitigating Cardiac Remodelling

Cardiac remodelling refers to a group of molecular, cellular and interstitial alterations that manifest clinically as changes in the size, shape structure, and function of the heart [114]. Celastrol exhibits protection against myocardial fibrosis, cardiac hypertrophy and dysfunction, thereby mitigating detrimental cardiac remodelling.

Excessive activity of RhoA/Rho-kinase (ROCK) pathway promotes the development of cardiovascular diseases, including HF [115]. Cyclophilin A (CyPA) and basigin (Bsg) play critical roles as downstream targets of ROCK in the enhancement of ROS production [116]. Celastrol was found by utilising high-throughput screening to reduce the expressions of CyPA and Bsg in the heart and the lung, alleviating pressure overload-induced cardiac dysfunction and postcapillary pulmonary hypertension (PH) [117]. Left ventricular HF is frequently accompanied by PH, negatively impacting symptoms, exercise capacity, and outcome [118]. As Cypa and Bsg activate NF-κB, the inhibitory effect of celastrol on CyPA/Bsg-NF-κB axis supports the notion that celastrol suppresses NF-κB activity to exert anti-inflammatory properties [119]. Furthermore, celastrol inhibits ROCK2 expression whilst sparing ROCK1. Since ROCK2 promotes cardiac hypertrophy and ROCK1 is cardioprotective, celastrol ameliorates pathological cardiac remodelling and postcapillary PH by blocking the corresponding augmentation of detrimental ROCK2 signalling. The same research group further explored the inhibitory effect of celastrol on CyPA and Bsg on right ventricular failure; the findings showed that celastrol reduced oxidative stress, inflammation, and abnormal proliferation in pulmonary artery smooth muscle cells, subsequently resulting in decreased pulmonary artery remodelling and alleviation of right ventricular failure. In hypoxia-induced PH in mice and SU5416/hypoxia-induced PH in rats, celastrol ameliorates right ventricular systolic pressure, hypertrophy, fibrosis and dysfunction via suppression of Bsg and CyPA [120].

Previous studies support the role of abnormally activated STAT3 in cardiac dysfunction [121]. Celastrol was recently reported to reduce angiotensin II-associated deleterious cardiac remodelling by inhibiting STAT3 activity [122]. In vitro experiments showed celastrol abolished STAT3 phosphorylation and nuclear translocation in rat primary cardiomyocytes and H9c2 cells, exerting the anti-hypertrophic and anti-fibrotic effects. Celastrol reduced the levels of β-myosin heavy chain (β-MHC), collagen I, and TGF-β1 in cells that were pretreated with celastrol before or after Ang II exposure. The chromatin immunoprecipitation qPCR assay demonstrated that celastrol blocked STAT3 by competitively direct binding to the promoter sites. In both angiotensin II-challenged and transverse aortic constriction (TAC)-stressed mice, celastrol treatment improved cardiac function, inhibited cardiac hypertrophy and fibrosis via inhibiting STAT3 [122].

Moreover, celastrol ameliorates myocardial fibrosis, left ventricular hypertrophy and cardiac dysfunction induced by TAC in mice via downregulating microRNA-21 (miR-21) and inhibiting the activated extracellular signal regulated kinases (ERK) [123]. In vitro study revealed that celastrol attenuated miR-21 upregulation by blocking the upstream TGF-β1 and decreased p-ERK/ERK levels in cultured cardiac fibroblasts.

In fructose-induced hypertensive rats, celastrol inhibited hypertension-induced vascular and cardiac hypertrophy [124]. Celastrol treatment also suppressed the circulating inflammatory cytokines such as TNF-α and IL-6, decreased ROS generation, lowered systolic and diastolic blood pressure, and blocked ERK/Akt activation via increasing HMOX-1 expression and activity [120,124]. HMOX-1 plays a crucial role in cytoprotective mechanisms including the maintenance of antioxidant/oxidant homeostasis and anti-inflammation [125,126].

### 6.2. Post-Ischaemic Cardioprotection

HF can develop as a complication of MI, thus it is imperative to preserve cardiac function as much as possible with post-MI treatment [127,128]. Pharmacological approaches that enhance the ischaemic conditioning or heat shock response (HSR) are of clinical significance in ameliorating myocardial I/R injury.

The central cardiac HSPs are HSP90 and HSP70 due to their regulatory roles in protein remodelling and inhibiting protein aggregation [129]. HSP90 is a master cellular homeostatic regulator in ischaemic conditioning. Inhibition of HSP90 expression disrupts ischaemic preconditioning in H9c2 cells, whereas overexpression of HSP90 protects against I/R stress in pig hearts [130]. Similarly, upregulation of HSP70 expression in response to I/R-induced aggregation of misfolded proteins produces cardioprotective effects by preventing cell apoptosis [131,132].

By disrupting the interaction of HSP90 and its co-factor CDC37 interaction, celastrol triggers powerful pro-survival signals, including HMOX-1, which is responsible for improved cardiac cell survival under hypoxic conditions. In the rat ischemic myocardium with permanent coronary ligation, continuous celastrol treatment for 14 days reduces infarct tissue size, improves cardiac function and abrogates adverse left ventricular remodelling [133]. The data suggest that celastrol displays cardioprotection dependent on ROS and HSF1 signalling. It is likely that the increase in oxidative stress after I/R triggers HSPs activation. Celastrol induces several HSP mRNA and protein expressions, including HSP70 and HMOX-1 (also known as HSP32), leading to the translocation of HSF1 protein from the cytoplasm to the nuclear fraction, the effect being abolished by a ROS inhibitor N-acetyl-L-cysteine (NAC) and an HSF1 inhibitor Kribb11. Another study is also in an agreement with the modulatory role of celastrol in the expression of HSP70 and HSP32 in H9c2 cells and ex vivo rat heart I/R model [133].

The work from the same group further reveals that celastrol as HSPs modulator protects against ischemia and prevents I/R injury by increasing the viability and reducing early mitochondrial permeability transition pore (mPTP) opening during reoxygenation [134]. The mPTP located in the inner membrane of the mitochondria is a critical mediator of myocardial I/R injury. It only opens during the first 2 to 3 min of reperfusion and keeps closed upon myocardial ischaemia. During reperfusion, the prolonged opening of mPTP results in mitochondrial swelling and the release of pro-apoptotic factors. Inhibition of mPTP opening at the onset of reperfusion has been linked to pre- and post-conditioning, where oxidative stress is reduced and reperfusion injury salvage kinase (RISK) signalling is activated [135,136].

Furthermore, celastrol suppresses myocardial apoptosis during I/R by activating PI3K/Akt and ERK1/2 kinases [133]. Akt and ERK1/2 belong to a group of pro-survival protein kinases involved in the cardioprotective RISK signalling triggered by ischaemic conditioning [137]. Another study also shows that celastrol pretreatment significantly decreases infarct size, as well as inhibiting myocardial apoptosis and oxidative stress via the activation of PI3K/Akt pathway and a reduction in high-mobility group box 1 protein (HMGB1) expression, a ubiquitous protein that induces apoptosis and pro-inflammatory cytokine release in I/R conditions [138].

Additionally, celastrol recovers cardiac function partly owing to its compelling anti-inflammatory property. An in vitro study using H9c2 cells showed that low-dose celastrol downregulated pro-inflammatory cytokines TNF-α and IL-1β and the transcription factor NF-κB at both mRNA and protein levels [139]. Similar observations were reported in the in vivo experiments that celastrol treatment markedly inhibited a range of pro-inflammatory cytokines including TNF-α, IL-1β, INF-γ and IL-6 expressions in I/R mouse and rat hearts [138,140].

## 7. Challenges of Celastrol and Strategies

Despite the tremendous advances in exploiting the pleiotropic modes of action, celastrol faces a few challenges, particularly its limited water solubility, low oral bioavailability, narrow therapeutic window of dosage, and potential side effects in vivo, which hamper its further clinical applications. Previous investigation of oral bioavailability and pharmacokinetics of celastrol in polyethanol glycol revealed poor systemic absorption in rats, as well as a short blood half-life of 8–10 h [141]. In a later study, celastrol was shown to possess low water solubility (13.25 ± 0.83 mg/mL at 37 °C), suggesting celastrol needs to be delivered with lipids such as olive oil or a self-micro emulsifying drug delivery system (SMEDDS) [142]. Another concern regarding the in vivo use of celastrol is its narrow window of dose. It was reported that the effective and non-toxic dosages of celastrol for the treatment of arthritis in rats are between 2.5 and 5 mg/kg/day, with lower concentrations being ineffective, while higher concentrations show signs of toxicity [143].

Regardless of celastrol being well tolerated in animal models overall, the possible detrimental side effects should be taken into consideration. The primary concern associated with celastrol administration is infertility. Studies have pointed out that celastrol concentration-dependently inhibited guinea pig sperm activity, which could be reversed after washing away celastrol within 3 h [144]. This inhibitory effect on spermatogenic cells was later suggested by inhibiting calcium currents [145]. Moreover, cardiac cytotoxicity was observed in the presence of high-dose celastrol [139,146]. Celastrol may also modulate resting metabolic rate and arterial pressure by increasing the sympathetic nerve activity of brown fat and kidney [19]. Apart from cardiovascular impact, the potential side effects of celastrol have been reported, including hepatotoxicity and nephrotoxicity [147,148]. Although celastrol shows certain common side effects, such as mild gastrointestinal disturbance, the overall toxicological profile of celastrol seems mild.

To improve the bioavailability and diminish the potential toxicity of celastrol, novel strategies with new delivery systems and the development of celastrol’s structural derivatives have been designed and tested. A recent study suggests that PEG-b-PPS micelles loaded celastrol is of great clinical interest owing to its enhanced bioavailability, high loading efficiency, and reduced cytotoxicity compared to free celastrol, where mice were well tolerated with continuous treatment of celastrol for 18 weeks [74]. Other strategies for the use of celastrol such as utilizing liposomes, SMEDDS, lipid nanospheres, and exosomes, were effective for treating various cancers [149,150,151]. More efficient delivery methods, especially nano-based carriers with conjugation of celastrol with receptor selective ligands to enhance absorption, specify the target organ or tissue, and minimize undesired effects are imperative. In addition, rational design of new celastrol derivatives or analogues with a better understanding of its structure–activity relationship is another effective strategy, and has been making good progress in recent years to further improve celastrol’s potent biological activities and simultaneously overcome its pharmacokinetics limitations and undesired side effects [3]. Obviously, the combination of currently developing strategies will be a plausible approach to offer more opportunities for the perspective clinical translation of celastrol in the future.

## 8. Conclusions and Perspectives

Discovering effective drugs for the prevention and treatment of CVD is an urgent but unmet clinical need. Given the fact that almost all CVD are multifactorial and systemic syndromes, and highly interrelated, effective therapeutic approaches are likely to require the targeting of these complex disorders in a more holistic manner. Over the last few decades, a large amount of research supports that celastrol has diverse encouraging effects, alleviating a range of CVD by interacting with general yet varied signalling pathways to provoke therapeutic effects. For example, oxidative stress and inflammation are critically involved in the initiation and progress of many CVD and their related comorbidities. As stated above, ample evidence shows that celastrol exerts wide cardiovascular benefits through its anti-oxidative and anti-inflammatory ability as common mechanisms. More significantly, ROS-generating protein NOX2 has emerged as especially important in cardiovascular pathologies, including obesity, diabetes, inflammation, atherosclerosis, valve calcification, vascular and cardiac remodelling, and heart failure [102]. As a potent while non-specific NOX2 inhibitor, celastrol therefore has unique advantages as an interesting and promising drug candidate for CVD through more combined and comprehensive actions, targeting not only directly cardiovascular central mechanisms, but also indirectly targeting peripheral systemic abnormalities.

Although lots of works have tried to illustrate the complex mechanisms responsible for pharmacological activities and phenotypic protection of celastrol, the precise molecular signalling pathways have not yet been fully identified, and the roles of some targets themselves are still unsettled. For instance, there seems to be conflict in that celastrol may either promote or prevent cell apoptosis to exert anti-tumour and cardiac protective effects [152,153], which could be related to the different action sites and dosages of celastrol used. However, based on the findings that celastrol has no tissue-specific selectivity, the exact cellular mechanisms of celastrol for cell viability warrant further examination [140]. In addition, STAT3 was recently discovered as a direct target by celastrol in AngII-induced cardiac dysfunction; however, previous studies suggest that the intriguing role of STAT3 may be context dependent. Thus, further work is needed to clarify the effectiveness by targeting STAT3 with celastrol in different disease settings [122,154]. Undoubtedly, celastrol has multiple pharmacological activities, identifying and targeting pivotal molecular pathways, especially the actions of celastrol in diverse CVD and at different stages of disease development will prove challenging, and may require more integrated application of state-of-the-art technologies, such as biochip, biosensing, network medicine analysis and multi-omics mapping, that generate large-scale transcriptional and protein expression signatures, to comprehensively understand the profiling preclinical mechanisms of celastrol. Indeed, the feasibility of this approach has successfully been demonstrated by the discovery of IL1R1 as a sensible target of celastrol’s anti-obesity effect with the use of transcriptomes [21].

In conclusion, celastrol has thus far exhibited significant potential as an attractive drug candidate for the treatment of CVD through complex but interlinked mechanisms, in particular, owing to its beneficial effects against oxidation and inflammation, actioning both locally and systemically. Further elimination of its pharmacokinetic limitations will be necessary to facilitate its future clinical trials. Therefore, additional research focusing on the novel delivery systems and development of celastrol’s structural derivatives need to be prioritized.

## Data Availability

Not applicable.
